# Organic Holmium(III) Complexes as a Potential Bright Emitter in Thin Films

**DOI:** 10.1021/acs.jpclett.2c02516

**Published:** 2022-10-21

**Authors:** Chen Lyu, Junjie Zhang, Filippo Boi, Huanqing Ye

**Affiliations:** † Institute of Fundamental and Frontier Sciences, University of Electronic Science and Technology of China, Chengdu610054, P.R. China; ‡ Photon Science Institute, Department of Electrical and Electronic Engineering, University of Manchester, ManchesterM13 9PY, United Kingdom; § College of Materials Science and Engineering, China Jiliang University, Hangzhou310018, P.R. China; ∥ College of Physics, Sichuan University, Chengdu610064, P.R. China

## Abstract

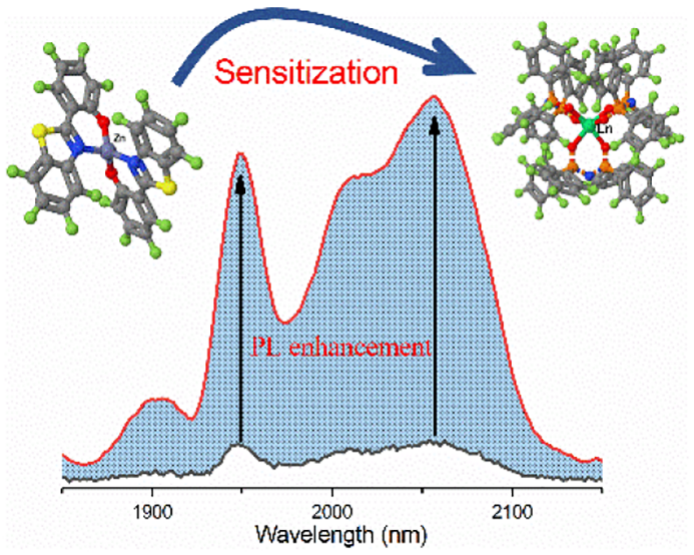

Holmium(III) ions incorporated with an organic ligand generate ∼2 μm optical emission which is characterized by steady and time-resolved photoluminescence. A potential efficient sensitization scheme is demonstrated by empirically calculating the Förster energy transfer rate and modeling the excited state dynamics of the ion. This is demonstrated by taking into account an ideal organic chromophore. The presented work proposes a promising material candidate for the 2 μm emission, which can be fabricated in thin films.

The 2 μm wavelength is favored for low propagation loss, attenuated photon energy, and large optical depth in water, which makes the light sources ideal in optical telecommunication, industrial laser fabrication, and ultrafast optical medical treatment.^1^ A typical 2 μm emitting material system is GaSb-based quantum dots, but their fabrication conditions are complicated because of the sensitivity of water.^1−5^ Another way of generating a 2 μm photon is via the 4f–4f transitions of lanthanide ions, Ho^3+^ and Tm^3+^ ions, which are narrow in line width and have relatively long lifetimes.^6−10^ Ho^3+^ and Tm^3+^ ions can be easily doped to glass hosts for the fabrication of 2 μm lasers and optical amplifiers, whereas these devices require high pump power to induce forbidden 4f transitions due to the low absorption coefficients and lack of integrability for advanced integrated technologies. Despite some sensitization schemes using other lanthanide ions to excite Ho^3+^ indirectly or Tm^3+^ ions, the enhancement of the emission intensity is still trivial with a small factor of <10.^11−16^ In this context, organic sensitized lanthanide materials provide a possible solution. In these systems, a light-harvesting organic chromophore efficiently sensitizes nearby lanthanide ions due to the coupling between organic excitons and lanthanide energy levels.^17−20^ However, the organics commonly have high-energy vibration bonds, including C–H, O–H, and N–H bonds, and they quench the lanthanide emission severely.^21,22^ The quenching effect becomes more challenging to eliminate when the lanthanide emission extends to a low energy region (*e.g*., long NIR wavelengths) because of the higher coupling grade of the vibration energy and the lanthanide transition energy. For example, measuring the 1.5 μm emission brightness of most organic erbium materials is challenging. Within numbers of those sensitization materials, a composite system of a chromophore, Zn(F-BTZ)_2_ and a perfluorinated organic complex, Ln(F-TPIP)_3_ (Ln = Nd, Er, and Yb) has a tremendous sensitization effect that provides the ion NIR emission with up to an enhancement factor of 10^4^ times.^23−25^ In this system that has Zn(F-BTZ)_2_ and Ln(F-TPIP)_3_ molecules mixed at the molecular level, the average separation of the two molecules is believed to be within a range of ∼1 to 2 nm, which is approximately the size of the molecule.^25−27^ Meanwhile, the perfluorinated organic environment and bulky ligand cage significantly protect the ions from the quenching effect. Dispite this success, the emitting region has only extended to an ∼1.5 μm band with an internal quantum efficiency of ∼30%.^21^ How longer wavelengths of a measurable lanthanide emission can be extended using this approach is an open question. In this work, we empirically investigate and discuss the performance of the 2 μm emission of Ho^3+^, which presumes that similar fabrication techniques can fabricate a Ho(F-TPIP)_3_ and Zn(F-BTZ)_2_ composite film and that the Ho^3+^ ions share comparable energy transfer mechanics with those other lanthanide ions.

A cage of F-TPIP^–^ ligands is used to coordinate a Ho^3+^ ion to eliminate vibrational quenching from external environments. The photoluminescence (PL), photoluminescence excitation (PLE), and time-resolved PL spectra of Ho(F-TPIP)_3_ powder are characterized to demonstrate the features of Ho^3+^ emission at the 2 μm band. A rate equation model is built to simulate the energy transfer process from Zn(F-BTZ)_2_ to Ho(F-TPIP)_3_. Zn(F-BTZ)_2_ refers to the conjugate base of 2-(tetrafluoro-2-hydroxphenyl) tetrafluorobenzothiazole. The spectroscopy of Zn(F-BTZ)_2_ has been well studied in previous research. Fitting the emission spectra of Zn(F-BTZ)_2_ to Förster calculations gives a singlet energy transfer rate of 9 ± 1.1(10^8^) s^–1^ and a triplet energy transfer rate of 900 ± 80 s^–1^. By feeding the energy transfer rate to the rate equation model, we obtain a potential PL enhancement of ∼8000 times, which exceeds any reported sensitization system in Ho^3+^ material systems.

The blue curve illustrates the emission spectrum of Ho^3+^ in Figure 1. A 440 nm pulsed Nd:YAG OPO laser pumps the Ho(F-TPIP)_3_ powder to excite the Ho^3+^ ions from ^5^F_1_ to ^5^G_6_ states. The excited states relax to the lowest excited state of ^5^I_8_ and decay back to the ground state ^5^I_8_ to give the emission band from 1850 to 2100 nm. Herein we exclude the contribution of the F-TPIP ligand to the emission peak as the energy gap does not match the emission energy at 2 μm. The excitation spectra shown by the red curve are recorded by monitoring the PL intensity at the wavelength of 2050 nm while the powder is pumped with wavelengths from 430 to 550 nm. The excitation peaks at 420 nm, 450 nm, 470 nm, 480 nm, and 490 nm are in good agreement with the Ho^3+^ absorption spectrum, representing the transition from ground state ^5^I_8_ to excited states of ^5^G_5,_^5^F_1_ + ^5^G_6_, ^3^K_8_, ^5^F_2_, and ^5^F_3_, respectively. The differences of the PLE intensities reflect the relative strength of the dipole oscillation for each transition.

**Figure 1:**
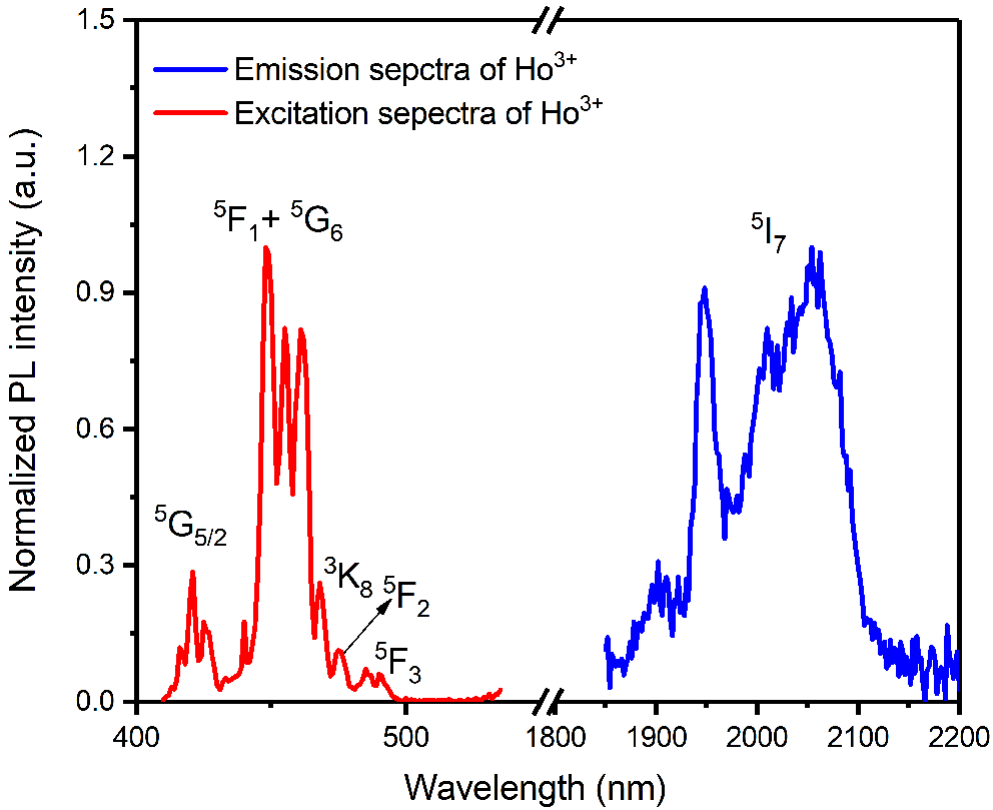
Spectra of Ho(F-TPIP)_3_ powder. The red curve indicates the PL excitation spectra at a monitoring wavelength of 2050 nm. The blue curve represents the emission spectrum with an excitation wavelength of 450 nm. The PL intensities are corrected with respect to the response of photon detectors and excitation power.

The time-resolved PL spectrum of the Ho(F-TPIP)_3_ powder at the wavelength of 2050 nm is shown in Figure 2. The spectrum is fitted with the combination exponential rising and exponential decay process, giving a fitted decay lifetime of 11 ± 0.7 μs and a rise of 7 ± 0.3 μs, respectively. The 7 μs rise time is an overall relaxation time for electrons at the high energy excited states to decay to the lowest excited state. This fast relaxation process indicates that most electrons accumulate at the lowest excited state before they decay back to the ground state. If we use the literature reported 3 ms lifetime as the intrinsic lifetime of Ho^3+^, an internal quantum yield of 0.3% is obtained from the powder sample. We assume that the lifetime of Ho^3+^ could be prolonged when it is mixed into a composite with Zn(F-BTZ)_2_ along with proper encapsulation techniques and diluters of some nonemissive organic complexes such as Y(F-TPIP)_3_.^28^ Also, further purification and waterproof encapsulation that suppress the nonradiative quenching effect from X–H (X = O, C, N, ...) bonds is a practical approach to improve quantum efficiency.

**Figure 2:**
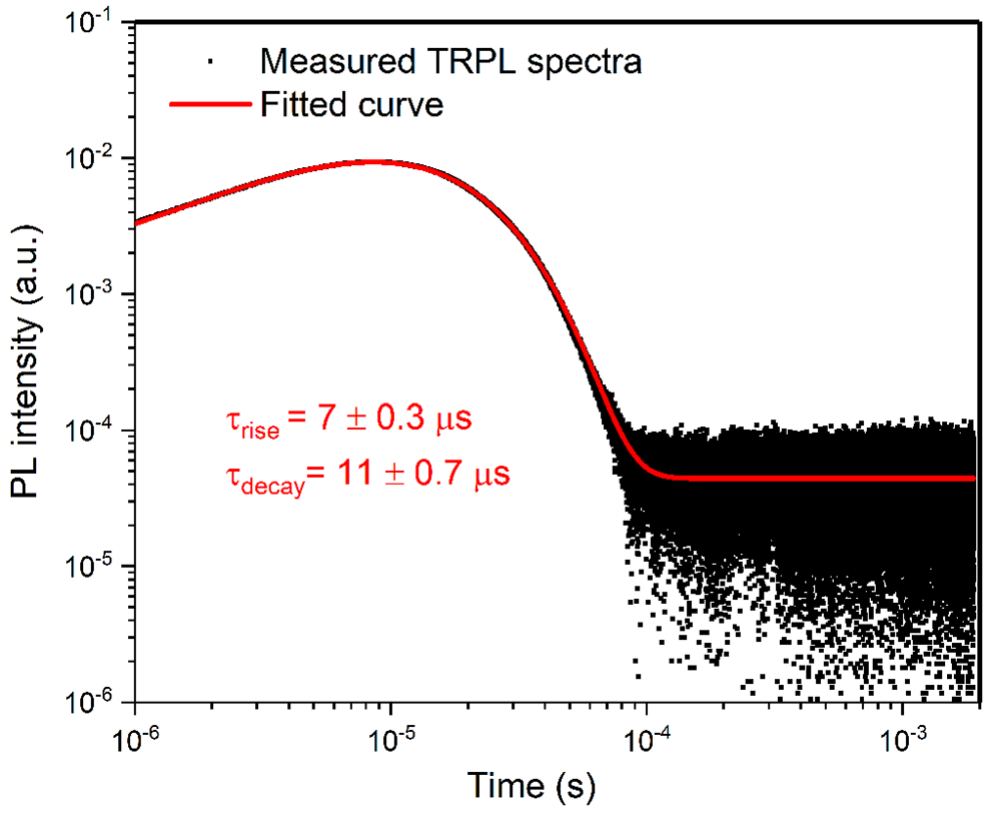
Time-resolved photoluminescence spectrum of Ho(F-TPIP)_3_ powder at 2050 nm. The powder is pumped by a 450 nm pulsed laser. The black dots suggest the measured data, and the red curve is the fitting results.

As the absorption spectra illustrate in Figure 3, Ho^3+^ ions have multiple absorption peaks in the visible range, providing energy transfer routes to sensitized Ho^3+^ ions with some light harvesting materials. The reason the PLE spectrum has more fine structures than the absorption spectrum is that the PLE process involves the internal relaxation processes coupled with possible multiphonon interactions within the excited states of Ho^3+^ ions. In this work, we take an organic chromophore Zn(F-BTZ)_2_ as a sensitizer to theoretically demonstrate the PL intensity enhancement of Ho^3+^ ions in an organic codoped system. The singlet and triplet emission spectrum of Zn(F-BTZ)_2_ is shown by the red and blue curves in Figure 3 in which the curves are normalized to the area of 1. As mentioned, Zn(F-BTZ)_2_ molecules should allocate nearby Ln(F-TPIP)_3_ molecules. The energy transfer from the singlet and triplet to the excited states of the Ho^3+^ ions can be predicted to be primarily driven by the Förster energy transfer mechanism. The Förster energy transfer rate can be quantified with the equations below:
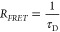
1
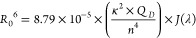
2
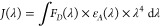
3

**Figure 3:**
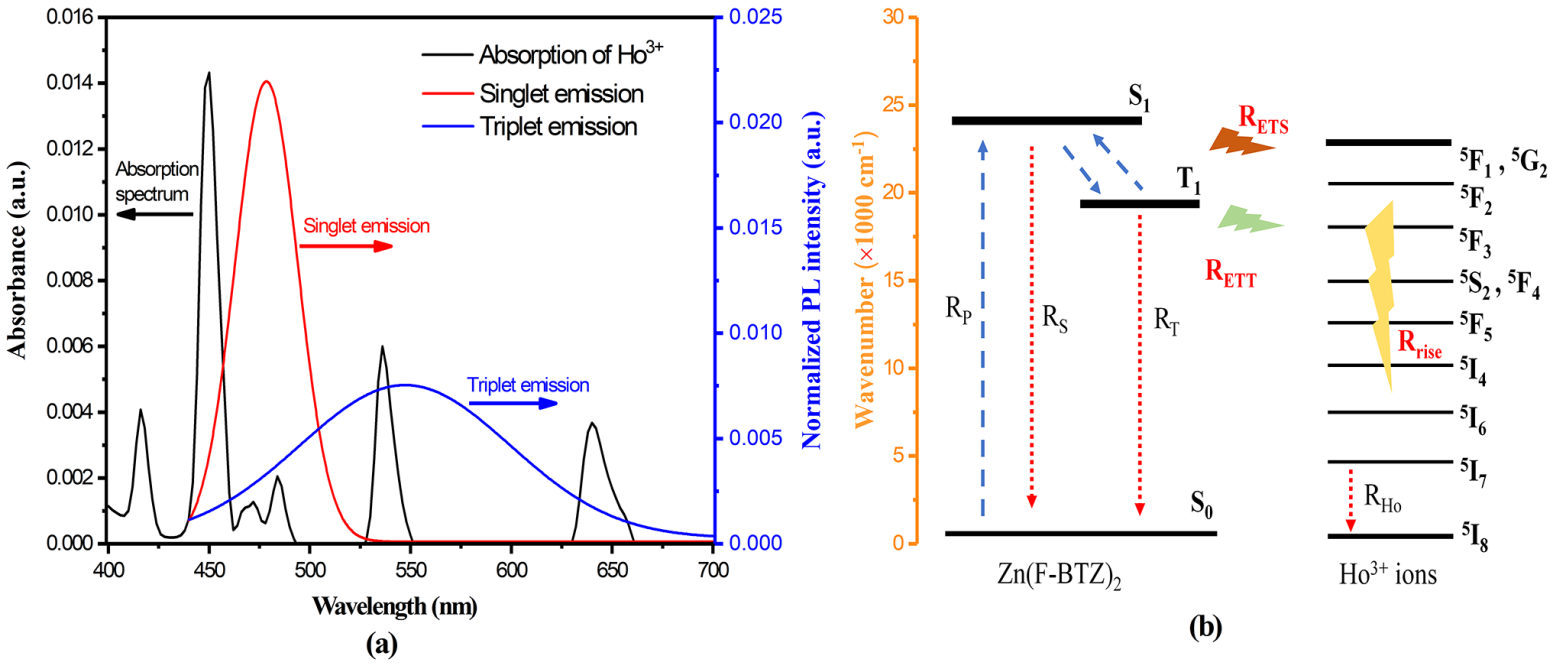
(a) Absorption spectrum of Ho^3+^ and emission spectra of the singlet and triplet of Zn(F-BTZ)_2_.^29^ (b) Energy transfer diagram of the sensitization process from Zn(F-BTZ)_2_ to Ho^3+^ ions.

In the equations above, *J*(*λ*) is the spectral overlap between the absorption spectra of Ho^3+^ and the emission spectra of Zn(F-BTZ)_2_. *Q_D_* is the quantum efficiency of the donner, where we use 0.3% for the triplet and 100% for the singlet. *κ*^2^ is a constant with a value of 2/3. Furthermore, *r* is the distance from the doner to the acceptor, and we use 7–10 Å in this paper, which is defined by the radii of Ln(F-TPIP)_3_ and Zn(F-BTZ)_2_.^26^ The calculated singlet and triplet energy transfer rates are ∼9 ± 1.1 (10^8^) s^–1^ and ∼900 ± 80 s^–1^, respectively.

According to the calculated energy transfer rate, the dynamics of the excited state population is analyzed by rate equation modeling. With the assumption that the system is pumped by a continuous wave to a steady state, the dynamics of the excited state population could be described by the equations below:
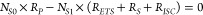
4
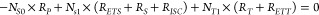
5
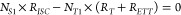
6
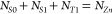
7
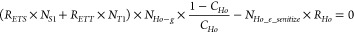
8
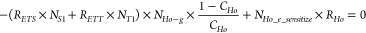
9
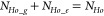
10
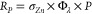
11

In eqs 4 to 7, *N*_*S*__0_*N*_*S*__1_, and *N*_*T*__1_ describe the dynamics of the ground state, the lowest singlet state, and the lowest triplet state of the Zn(F-BTZ)_2_ chromophore. Their sum equals the total number of Zn(F-BTZ)_2_ chromophores in the film. *R*_*S*_, *R*_*T*_, *R*_*ISC*_, *R*_*ETS*_, and *R*_*ETT*_ indicate the singlet decay rate, triplet decay rate, intersystem crossing rate, singlet energy transfer rate, and triplet energy transfer rate. Indicated by eqs 8 to 10, the population of the singlet and triplet states, *N*_*S*__1_ and *N*_*T*__1_, determines the number of Ho^3+^ ions that can be sensitized. The terms *N*_*Ho_g*_ and *N*_*Ho_e_sensitize*_ give the population in the Ho^3+^ ground state and the lowest excited state. *N*_*HO*_ and *R*_*HO*_ represent the total number of Ho^3+^ ions and its decay rate. *C*_*HO*_ indicates the concentration of Ho(F-TPIP)_3_.

Since the relaxation rate from the Ho^3+^ ions’ excited state is swift, we approximate the energy state of Ho^3+^ to a two-level system. Hence, the rate equations used to simulate the direct excitation process are shown below.
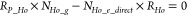
12
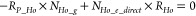
13
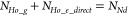
14
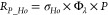
15

In the equations above, *R*_*P_Ho*_ indicates the direct excitation rate, which could be calculated with the absorption cross-section of Ho^3+^. It is noteworthy that the lifetime of Ho^3+^ only determines the quantum efficiency at the lowest excited states. Also, we consider that there is no back energy transfer from Ho^3+^ to Zn(F-BTZ)_2_, which allows us to assume that the radiative rate of Ho^3+^ remains the same when the film is excited via either sensitization or direct excitation. Hence, the PL intensity enhancement can be given by Equation 16:
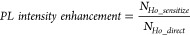
16

In a sensitization process, both triplet and singlet states sensitize the excited state of Ho^3+^ with a triplet energy transfer rate *R_ETT_* and a singlet energy transfer rate *R_ETS_*. Whereas, at room temperature, the quantum efficiency of the triplet state is extremely low due to thermal quenching, which only builds a small triplet population for transfer energy. Thus, in this modeling, we only investigate the influence of *R_ETS_* on the sensitized PL intensity enhancement. It is worth noting that the triplet energy transfer process could be enhanced by reducing the temperature to suppress the thermally activated processes such as triplet–triplet annihilation and reversed intersystem crossing.

Considering the excited state density of Ho^3+^ under sensitization and direct excitation for the PL intensity, we use eqs 1–16 to simulate the sensitized PL intensity and PL intensity enhancement, which are shown in Figure 4a,b. The details of the simulation method, including Mathematica programming, codes, and numerical calculations, are presented in the SI. Given a concentration of 10% and a *R_ETS_* equal to 9 (10^8^) s^–1^, with the increase in pump power density, the population at the excited state of Ho(F-TPIP)_3_ is saturated at a high pump power density. The reason the lowest doping concentration has the brightest sensitized PL intensity is because the low quantum efficiency of the Ho^3+^ quenches most of the excited state population. That suggests even when the sensitization process is designed to be efficient by creating a large spectral overlap between the lanthanide ions and the chromophore, the lanthanide ions still need to have a significant quantum efficiency to give bright light emission.

**Figure 4:**
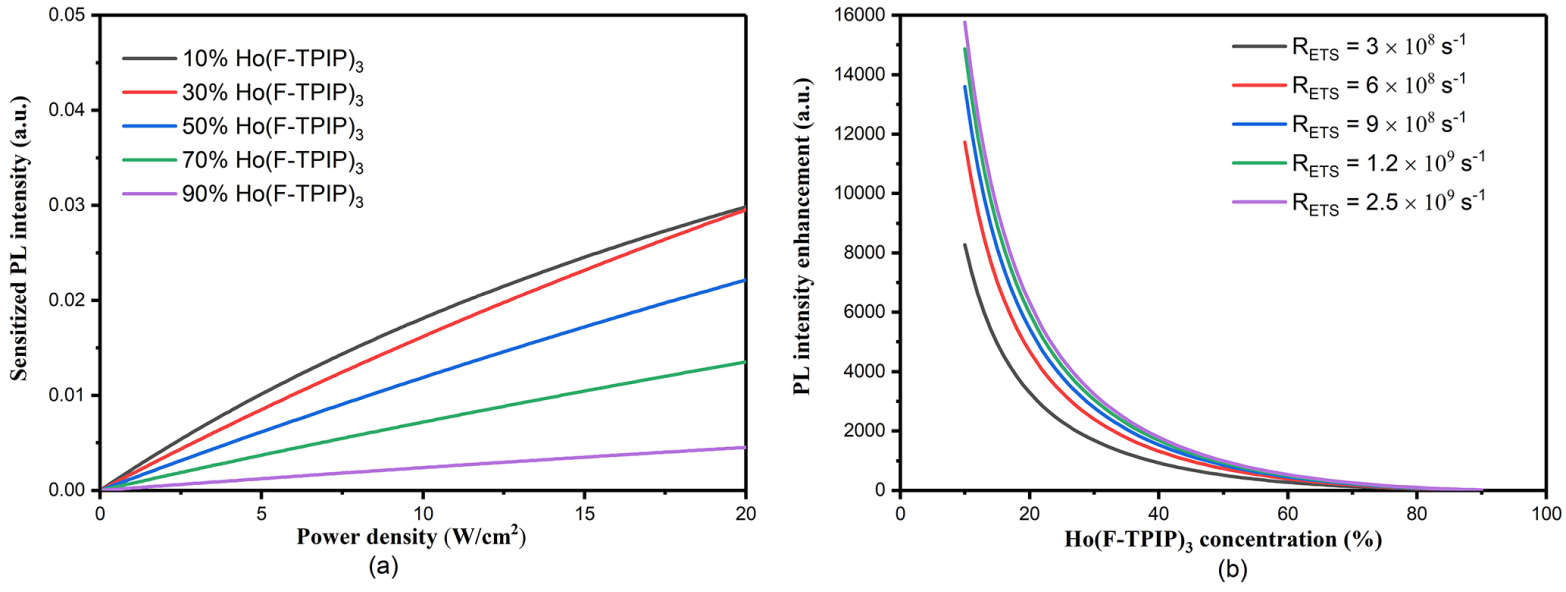
Simulated PL intensity enhancement and sensitized PL intensity. (a) PL intensity enhancement with the increase of Ho(F-TPIP)_3_ concentration with different *R*_ETS_ values. (b) Sensitized PL intensity with power dependence for different doping concentrations.

Illustrated by the red curve in Figure 4b, with the calculated *R_ETS_* of 9 (10^8^) s^–1^, the PL enhancement could reach 12 000 times compared to direct excitation with a Ho(F-TPIP)_3_ concentration of 10%. Increasing the doping concentration would reduce the PL intensity enhancement due to the decreased excitation probability for individual Ho^3+^ ions. Herein, we validate our simulation by considering the uncertainties of the parameters we use in this model. The most significant simulation error comes from the Förster energy transfer rate calculation that is introduced by the uncertainty of the donor–acceptor distance. If we give a range of separation distances from 7 to 10 Å, the calculated singlet energy transfer rate would vary form 2.9 (10^8^) s^–1^ to 2.5 (10^9^) s^–1^. In such a case, the maximum PL intensity enhancement at a low Ho(F-TPIP)_3_ concentration increases from 8000 to 16 000 times. That strongly indicates that a potential efficient energy transfer route exists between Ho^3+^ and Zn(F-BTZ)_2_. Even with the lowest *R_ETS_* from the Förster calculation, the simulation still gives 8000 times PL intensity enhancement, which is three magnitudes larger than the current reported sanitization schemes in the inorganic material system.

This work demonstrates the 2 μm emission from a perfluorinated Ho^3+^ complex, Ho(F-TPIP)_3_. The PLE and TRPL spectra of the Ho(F-TPIP)_3_ powder are presented to give a perspective of the luminescence property of Ho^3+^. A theoretical model predicts the efficiency energy coupling between a light-harvesting organic chromophore Zn(F-BTZ)_2_ and Ho^3+^ ions, which may yield ∼8000 to 16 000 times PL intensity enhancement via the sensitization process. Incorporating Zn(F-BTZ)_2_ and Ho(F-TPIP)_3_ in thin films opens a new opportunity to fabricate integrated light emitting devices at 2 μm.

## Experimental Method

The synthesis of the organic complex Ho(F-TPIP)_3_ can be found in the Supporting Information. The crude Ho(F-TPIP)_3_ is purified by a train vacuum purification system before performing any optical characterization. The PL signals are collected by a Triax 550 spectrometer and detected by a Hamamatsu R5509-72 nitrogen cooled photomultiplier.

## Supporting Information Available

Chemical properties and synthesis procedure of Ho(F-TPIP)_3_ and the parameters for the Förster energy transfer calculation (PDF)

## References

[ref1] Scholle K.; Lamrini S.; Koopmann P.; Fuhrberg P. 2 μm Laser Sources and Their Possible Applications. In Frontiers in guided wave optics and optoelectronics; IntechOpen: 2010.

[ref2] Zia N.; Viheriälä J.; Koskinen R.; Koskinen M.; Suomalainen S.; Guina M. Fabrication and Characterization of Broadband Superluminescent Diodes for 2 μm Wavelength. In Light-Emitting Diodes: Materials, Devices, and Applications for Solid State Lighting XX; SPIE: 2016; Vol. 9768, pp 71–79.

[ref3] Rösener B.; Rattunde M.; Moser R.; Kaspar S.; Manz C.; Köhler K.; Wagner J. GaSb-Based Optically Pumped Semiconductor Disk Lasers Emitting in the 2.0–2.8 μm Wavelength Range. In Solid State Lasers XIX: Technology and Devices; SPIE: 2010; Vol. 7578, pp 250–257.

[ref4] Hopkins J.-M.; Hempler N.; Rösener B.; Schulz N.; Rattunde M.; Manz C.; Köhler K.; Wagner J.; Burns D. High-Power,(AlGaIn)(AsSb) Semiconductor Disk Laser at 2.0 μm. Opt. Lett. 2008, 33 (2), 201–203. 10.1364/OL.33.000201. 18197239

[ref5] Rattunde M.; Schmitz J.; Mermelstein C.; Kiefer R.; Wagner J. III-Sb-Based Type-I QW Diode Lasers. Mid-infrared Semicond. Optoelectron. 2006, 118, 131–157. 10.1007/1-84628-209-8_3.

[ref6] Li N.; Magden E. S.; Su Z.; Singh N.; Ruocco A.; Xin M.; Byrd M.; Callahan P. T.; Bradley J. D. B.; Baiocco C.; et al. Broadband 2-μm Emission on Silicon Chips: Monolithically Integrated Holmium Lasers. Opt. Express 2018, 26 (3), 2220–2230. 10.1364/OE.26.002220. 29401762

[ref7] Fan X.; Kuan P.; Li K.; Zhang L.; Li D.; Hu L. Spectroscopic Properties and Quenching Mechanism of 2 μm Emission in Ho^3+^ Doped Germanate Glasses and Fibers. Opt. Mater. Express 2015, 5 (6), 1356–1365. 10.1364/OME.5.001356.

[ref8] Tian Y.; Zhang L. Y.; Xu R. R.; Hu L. L.; Zhang J. J. 2 μm Emission Properties in Tm^3+^/Ho^3+^ Codoped Fluorophosphate Glasses. Appl. Phys. B: Laser Opt. 2010, 101 (4), 861–867. 10.1007/s00340-010-4142-3.

[ref9] Zhang W. J.; Zhang Q. Y.; Chen Q. J.; Qian Q.; Yang Z. M.; Qiu J. R.; Huang P.; Wang Y. S. Enhanced 2.0 μm Emission and Gain Coefficient of Transparent Glass Ceramic Containing BaF_2_: Ho^3+^, Tm^3+^ Nanocrystals. Opt. Express 2009, 17 (23), 20952–20958. 10.1364/OE.17.020952. 19997333

[ref10] Kochanowicz M.; Zmojda J.; Miluski P.; Baranowska A.; Leich M.; Schwuchow A.; Jäger M.; Kuwik M.; Pisarska J.; Pisarski W. A.; et al. Tm ^3+^/Ho^3+^ Co-Doped Germanate Glass and Double-Clad Optical Fiber for Broadband Emission and Lasing above 2 μm. Opt. Mater. Express 2019, 9 (3), 1450–1458. 10.1364/OME.9.001450.

[ref11] Bai G.; Guo Y.; Tian Y.; Hu L.; Zhang J. Light Emission at 2 μm from Ho-Tm-Yb Doped Silicate Glasses. Opt. Mater. (Amst). 2011, 33 (8), 1316–1319. 10.1016/j.optmat.2011.03.033.

[ref12] Chen R.; Tian Y.; Li B.; Jing X.; Zhang J.; Xu S.; Eckert H.; Zhang X. Thermal and Luminescent Properties of 2 μm Emission in Thulium-Sensitized Holmium-Doped Silicate-Germanate Glass. Photonics Res. 2016, 4 (6), 214–221. 10.1364/PRJ.4.000214.

[ref13] Tian Y.; Zhang L.; Feng S.; Xu R.; Hu L.; Zhang J. 2 μm Emission of Ho^3+^-Doped Fluorophosphate Glass Sensitized by Yb^3+^. Opt. Mater. (Amst). 2010, 32 (11), 1508–1513. 10.1016/j.optmat.2010.06.012.

[ref14] Wang M.; Yu C.; He D.; Feng S.; Li S.; Zhang L.; Zhang J.; Hu L. Enhanced 2 μm Emission of Yb-Ho Doped Fluorophosphates Glass. J. Non. Cryst. Solids 2011, 357 (11–13), 2447–2449. 10.1016/j.jnoncrysol.2010.11.073.

[ref15] Wang M.; Yi L.; Wang G.; Hu L.; Zhang J. 2μm Emission Performance in Ho^3+^ Doped Fluorophosphate Glasses Sensitized with Er^3+^ and Tm^3+^ under 800 nm Excitation. Solid State Commun. 2009, 149 (29–30), 1216–1220. 10.1016/j.ssc.2009.04.021.

[ref16] Xu R.; Tian Y.; Hu L.; Zhang J. Efficient∼ 2 μm Emission and Energy Transfer Mechanism of Ho^3+^ Doped Barium Gallium Germanate Glass Sensitized by Tm^3+^ Ions. Appl. Phys. B: Laser Opt. 2012, 108 (3), 597–602. 10.1007/s00340-012-5113-7.

[ref17] Li H.; Liu X.; Lyu C.; Ma F.; Ye H.; Wyatt P. B.; Gillin W. P. Enhanced 1.54 μm Luminescence of a Perfluorinated Erbium Complex Sensitized by Perfluorinated Pt (II) and Zn (II) Phthalocyanines with 980 Nm Emission. J. Mater. Chem. C 2021, 9 (2), 456–465. 10.1039/D0TC05301E.

[ref18] Wei H.; Yu G.; Zhao Z.; Liu Z.; Bian Z.; Huang C. Constructing Lanthanide [Nd (III), Er (III) and Yb (III)] Complexes Using a Tridentate N, N, O-Ligand for near-Infrared Organic Light-Emitting Diodes. Dalt. Trans. 2013, 42 (24), 8951–8960. 10.1039/c3dt50778e. 23665838

[ref19] Quochi F.; Artizzu F.; Saba M.; Cordella F.; Mercuri M. L.; Deplano P.; Loi M. A.; Mura A.; Bongiovanni G. Population Saturation in Trivalent Erbium Sensitized by Organic Molecular Antennae. J. Phys. Chem. Lett. 2010, 1 (1), 141–144. 10.1021/jz900081m.

[ref20] Hernández I.; Gillin W. P. Organic Chromophores-Based Sensitization of NIR-Emitting Lanthanides: Toward Highly Efficient Halogenated Environments. In Handbook on the Physics and Chemistry of Rare Earths; Elsevier: 2015; Vol. 47, pp 1–100.

[ref21] Ye H. Q.; Peng Y.; Li Z.; Wang C. C.; Zheng Y. X.; Motevalli M.; Wyatt P. B.; Gillin W. P.; Hernández I. Effect of Fluorination on the Radiative Properties of Er^3+^ Organic Complexes: An Opto-Structural Correlation Study. J. Phys. Chem. C 2013, 117 (45), 23970–23975. 10.1021/jp4093282.

[ref22] Quochi F.; Orru R.; Cordella F.; Mura A.; Bongiovanni G.; Artizzu F.; Deplano P.; Mercuri M. L.; Pilia L.; Serpe A. Near Infrared Light Emission Quenching in Organolanthanide Complexes. J. Appl. Phys. 2006, 99 (5), 053520 10.1063/1.2177431.

[ref23] Ye H. Q.; Li Z.; Peng Y.; Wang C. C.; Li T. Y.; Zheng Y. X.; Sapelkin a; Adamopoulos G.; Hernández I.; Wyatt P. B.; Gillin W. P. Organo-Erbium Systems for Optical Amplification at Telecommunications Wavelengths. Nat. Mater. 2014, 13 (4), 382–386. 10.1038/nmat3910. 24651429

[ref24] Lyu C.; Li H.; Wyatt P. B.; Gillin W. P.; Ye H. Prolonged and Efficient Near-Infrared Photoluminescence of a Sensitized Organic Ytterbium-Containing Molecular Composite. J. Mater. Chem. C 2020, 8 (28), 9502–9505. 10.1039/D0TC02075C.

[ref25] Lyu C.; Li H.; Zhou S.; Liu G.; Wyatt P. B.; Gillin W. P.; Ye H. Bright and Efficient Sensitized Near-Infrared Photoluminescence from an Organic Neodymium-Containing Composite Material System. J. Am. Chem. Soc. 2021, 143 (43), 17915–17919. 10.1021/jacs.1c06827. 34676770

[ref26] Glover P. B.; Bassett A. P.; Nockemann P.; Kariuki B. M.; Van Deun R.; Pikramenou Z. Fully Fluorinated Imidodiphosphinate Shells for Visible- and NIR-Emitting Lanthanides : Hitherto Unexpected Effects of Sensitizer Fluorination on Lanthanide Emission Properties. Chem.—Eur. J. 2007, 13 (22), 6308–6320. 10.1002/chem.200700087. 17570719

[ref27] Li Z.; Dellali A.; Malik J.; Motevalli M.; Nix R. M.; Olukoya T.; Peng Y.; Ye H.; Gillin W. P.; Hernández I.; Wyatt P. B. Luminescent Zinc(II) Complexes of Fluorinated Benzothiazol-2-yl Substituted Phenoxide and Enolate Ligands. Inorg. Chem. 2013, 52 (3), 1379–1387. 10.1021/ic302063u. 23317157

[ref28] Hu J.; Wyatt P. B.; Gillin W. P.; Ye H. Continuous Tuning of Organic Phosphorescence by Diluting Triplet Diffusion at the Molecular Level. J. Phys. Chem. Lett. 2018, 9 (8), 2022–2024. 10.1021/acs.jpclett.8b00673. 29617138

[ref29] Huang F.; Li X.; Liu X.; Zhang J.; Hu L.; Chen D. Sensitizing Effect of Ho^3+^ on the Er^3+^: 2.7 μm-Emission in Fluoride Glass. Opt. Mater. (Amst). 2014, 36 (5), 921–925. 10.1016/j.optmat.2013.12.031.

