# Supplementary material for: Organic Holmium(III) Complexes as a Potential Bright Emitter in Thin Films
Source: J Phys Chem Lett. 2022 Oct 21;13(43):10101–6. doi: 10.1021/acs.jpclett.2c02516 (PMC9639195; doi:10.1021/acs.jpclett.2c02516)
Supplement: Supplementary file 1 — jz2c02516_si_001.pdf [file jz2c02516_si_001.pdf]

Supporting Information

Organic Holmium (III) Complexes as a Potential Bright Emitter in Thin Films

Chen Lyu[a], Junjie Zhang[c], Filippo Boi[d], and Huanqing Ye[b*]

[a] Institute of Fundamental and Frontier Sciences, University of Electronic Science and Technology of China, Chengdu, 610054, P.R. China

[b] Photon Science Institute, Department of Electrical and Electronic Engineering, University of Manchester, Manchester, M13 9PY, United Kingdom

[c] College of Materials Science and Engineering, China Jiliang University, Hangzhou, 310018, P.R. China

[d] College of Physics, Sichuan University, Chengdu, 610064, P.R. China

[*] Email: huanqing.ye@manchester.ac.uk

1. Material and method

1.1 Ho(F-TPIP)$_3$

Trivalent lanthanide ions Ho^{3+} are incorporated with three tetrakis-(pentafluorophenyl) imidodiphosphinate, HF-TPIP ligands to form the chelating organic complexes Ho(F-TPIP)$_3$.[1] Figure S1 shows the molecular structures of this series of lanthanide complexes. Each of these ligands contains four fully fluorinated phenyl rings. When three F-TPIP^{-1} ligands are bonded with one lanthanide ion, those phenyl rings form a sphere structure to isolate the lanthanide core from the quenching centres in the environment.

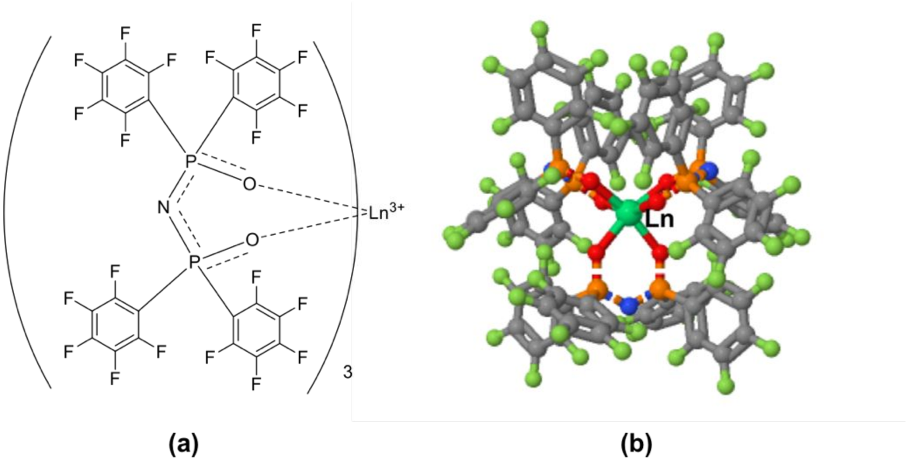

Figure S1. Molecule structure of Ln(F-TPIP)$_3$. (a) 2D structure of Ho(F-TPIP)$_3$. (b) 3D structure of Ho(F-TPIP)$_3$. Green balls indicate F atoms, red ball represent O atoms, orange balls are P atoms and blue balls correspond to N atoms. It's noteworthy that the molecular structure in the figure varies with different lanthanide ions due to the different atom size.

To synthesis $Ho(F\text{-}TPIP)_3$ complexes $HoCl_3{\cdot}6H_2O$ salt and HF-TPIP ligand are dissolved and reacted in boiling methanol and ethanol solution (1:1). Precipitates from the reaction are filtered out from the solution and dried in a vacuum oven at ~100 ˚C for 12 hours. The chemical equation to produce $Ln(F\text{-}TPIP)_3$ precipitation is shown by equation S1 where *Ln* indicates Ho.

$$HoCl_3{\cdot}6H_2O + 3H\text{-}FTPIP \xrightarrow{Boiling\ solution} Ho(F\text{-}TPIP)_3 + 3HCl + 6H_2O \quad \text{(S1)}$$

1.2 $Zn(F\text{-}BTZ)_2$

A well-studied organic chromophore, a zinc salt of 2-(tetrafluoro-2-hydroxyphenyl) tetrafluorobenzothiazole, $Zn(F\text{-}BTZ)_2$, is employed as an energy donor to sensitise $Ho(F\text{-}TPIP)_3$ complexes. The chemical formula and molecular structure of $Zn(F\text{-}BTZ)_2$ are shown in Figure S2. The conjugated π bonds within the molecules allows the intermolecular transportation of the delocalised electrons, which gives the material organic semiconducting properties. DFT calculation indicates that the HOMO and LUMO of $Zn(F\text{-}BTZ)_2$ chromophore is -6.14 eV and -2.65 eV, respectively.[2]

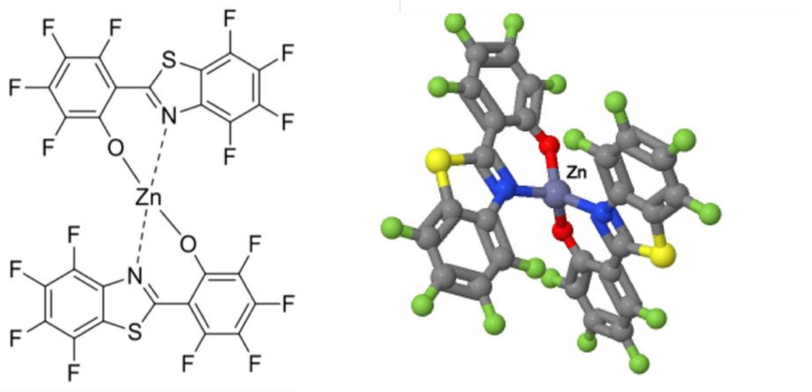

(a) (b)

Figure S2 Molecule structure of $Zn(F\text{-}BTZ)_2$ (a) 2D molecule structure (b) 3D molecule structure. Green balls are F atoms, red balls indicate O atoms, yellow balls represent S atoms and blue balls suggest N atoms.

2. Parameters for Förster resonant energy transfer (FRET) rate calculation

Parameter	τ_D	κ^2	n	$J(\lambda)$
Values for R_{ETT}	0.3 ± 0.05 s	2/3	1.47	$1.3 \pm 0.7 \times 10^{13}$ Å^6
Values for R_{ETS}	2 ± 0.3 ns	2/3	1.47	$6.8 \pm 0.4 \times 10^{12}$ Å^6

2. Methods for simulation

The simulation for the exciton density of singlet and triplet and the excited state population of Ho^{3+} ions are run by Wolfram Mathematica. The code of simulation is shown in Figure S3. As for the HOMO and LUMO information and the Contour Plot of $Zn(F\text{-}BTZ)_2$, they are presented in the

previous published paper.[2] Readers could refer to the 'Computational Studies' on page 1384 and Figure 11 for calculation details and the Contour Plot.

```
(* Assign parameters for calculation *)
Rs = 5 * 10^9; (* siglet decay rate *)
Rt = 157; (* triplet decay rate *)
Rho = 1 / (1.16 * 10^-5); (* Holmium decay rate *)
Risc = 3 * 10^7; (* Intersystem crossing rate *)
Ret = 6 * 10^10 (* energy transfer rate rate *)

(*Solve the steady state rate equation for singlet, triplet and Holmium's first excited state population*)
s = Solve[{((1 - c) - NS - NT) * 341 * P - NS * (Risc + Rets + Rs) == 0 , NS * Risc - NT * (Rett + Rt) == 0,
   ((1 - c) / c) * (NS * Rets + NT * Rett) * (c / (1 - c) - NHo) - NHo * Rnd == 0}, {NS, NT, NHo}]

Ho = NHo /. s // FullSimplify

PHo = Ho[[1]] (*sensitized Holmium excited state population*)
(*Solve the steady state rate equation for Holmium excited state under direct excitation*)

s2 = Solve[(c / (1 - c) - NHo1) * P * 0.128 - NHo1 * Rho == 0, NHo1]

Ho1 = NHo1 /. s2 // FullSimplify

PHo2 = Ho1[[1]] (* directly excited Holmium excited state population*)

PLe = Pho2 / Pho1 /. P → 1 (*PL intesnity enhancement under certain pump power density*)

Plot[Ple, {c, 0.1, 0.9}]
Plot[PHo2, {p, 0, 10}]
```

Figure S3. Mathematica code for simulation. The results of simulation are hidden for clarity.

References

(1) Glover, P. B.; Bassett, A. P.; Nockemann, P.; Kariuki, B. M.; Deun, R. Van; Pikramenou, Z. Fully Fluorinated Imidodiphosphinate Shells for Visible- and NIR-Emitting Lanthanides : Hitherto Unexpected Effects of Sensitizer Fluorination on Lanthanide Emission Properties. *Chem. Eur. J.* **2007**, *13* (22), 6308–6320.

(2) Li, Z.; Dellali, A.; Malik, J.; Motevalli, M.; Nix, R. M.; Olukoya, T.; Peng, Y.; Ye, H.; Gillin, W. P.; Hernández, I.; Wyatt, P. B. Luminescent Zinc(II) Complexes of Fluorinated Benzothiazol-2-yl Substituted Phenoxide and Enolate Ligands. *Inorg. Chem.* **2013**, *52* (3), 1379–1387.
